# Differences in Dry Sliding Wear Behavior between Al–12Si–CuNiMg Alloy and Its Composite Reinforced with Al_2_O_3_ Fibers

**DOI:** 10.3390/ma12111749

**Published:** 2019-05-29

**Authors:** Qing Zhang, Jie Gu, Shuo Wei, Ming Qi

**Affiliations:** 1 College of Engineering, China Agricultural University, Beijing 100083, China; 2 Beijing Institute of Space Launch Technology, Beijing 100076, China; ltt009x@gmail.com (J.G.); 18910189880@189.cn (S.W.); qm6814@163.com (M.Q.)

**Keywords:** sliding wear, Al_2_O_3_ fiber, aluminum matrix composite, friction

## Abstract

The dry sliding wear behavior of the Al-12Si-CuNiMg matrix alloy and its composite reinforced with Al_2_O_3_ fibers was investigated using a pin-on-disk wear-testing machine. The volume fraction of Al_2_O_3_ fibers in the composite was 17 vol.%. Wear tests are conducted under normal loads of 2.5, 5.0, and 7.5 N, and sliding velocities of 0.25, 0.50, and 1.0 m/s. Furthermore, the worn surfaces of the matrix alloy and the composite were examined using scanning electron microscopy (SEM) and energy dispersive spectroscopy (EDS). The results showed that the wear resistance of the composite was inferior to that of the matrix alloy, which could be attributed to the high content of reinforcement and casting porosities in the composite. Worn-surface analysis indicates that the dominant wear mechanisms of both materials were abrasive wear and adhesive wear under the present testing conditions.

## 1. Introduction

Cast aluminum alloys are widely utilized in the global automotive and aerospace industries due to their high specific strength and stiffness, low density, and excellent casting properties [1,2,3]. In recent years, aluminum matrix composites reinforced with discontinuous ceramic particles, fibers, or whiskers have been attracting extensive attention [4,5,6,7]. With the addition of ceramic reinforcement, the composites exhibit superior mechanical strength, thermal physical properties, and fatigue resistance contrasted with matrix alloys [8,9,10,11,12,13,14,15]. Regarding wear behavior, however, there is enormous difficulty in determining whether the reinforced composite or the matrix alloy possesses better wear resistance, since conflicting results exist in the published literature [16].

The wear behavior of the aluminum matrix alloy and its composite is affected by many factors. The principal factors that control wear performance can be classified into two categories, i.e., material factors and mechanical–physical factors [17]. Material factors, mainly including the reinforcement type and size, reinforcement volume fraction, matrix microstructure, and internal defects, have been investigated by many researchers. It was found that the improvement of wear resistance is strongly dependent on the type of reinforcement. The particle-reinforced composite exhibits the best wear resistance among aluminum matrix composites [7]. In addition, it was reported that the wear resistance of aluminum matrix composites is enhanced with increasing reinforcement contents and sizes [18,19,20]. However, conflicting results were also noted when the volume fraction of reinforcement exceeds a certain value [3,4,21]. Furthermore, with regard to the cast aluminum alloy and its composites, the influence of casting porosity on wear behavior cannot be ignored. Abdulhaqq et al. investigated the effect of casting porosity on the wear behavior of a fiber-reinforced aluminum matrix composite [22]. The results indicate that wear rate significantly increases with increasing casting porosities. The existence of casting porosity increases the real contact area within the counterface and promotes subsurface cracking and delamination. On the other hand, mechanical–physical factors, such as sliding velocity and normal load, have different effects on wear behavior. It was found that a higher transition load from mild wear to severe wear exists for the composite [23]. In addition, sliding velocity affects the wear behavior of materials by inducing friction heat and interface oxidation. The generated oxidation on the counterface can act as solid lubricant film or peeling-off layer, depending on the magnitude of sliding distance and normal load [20]. Generally, in actual operating conditions for many components, such as engine pistons, applied load and sliding velocity significantly vary during the operating cycle [24].

Under the aforementioned influence of material factors and mechanical–physical factors, the wear behavior of the aluminum matrix alloy and its composite is complicated. It is difficult to estimate which one possesses superior wear resistance, especially when the composite is not developed with a specific focus on improving its wear resistance. Therefore, in investigating the wear behavior of a specific aluminum alloy and its composite, it is better to carry out wear tests under various sliding velocities and loads, with the benefits of making a comparison between the two materials and exploring the influence of sliding velocity and load.

In previous studies, a cast Al–12Si–CuNiMg alloy and its composite reinforced with Al_2_O_3_ fibers were developed [25,26,27]. They exhibit excellent mechanical strength and fatigue resistance, especially under high-temperature conditions [28]. In the present work, the objective was to investigate the dry sliding wear behavior of both materials by conducting wear experiments over a range of sliding velocities and loads. The effect of sliding velocity and load on wear rate and friction coefficient was discussed in detail. In order to investigate the wear mechanisms, the worn surfaces of both materials were examined with the assistance of scanning electron microscopy (SEM) and energy dispersive spectroscopy (EDS).

## 2. Materials and Experimental Procedures

### 2.1. Materials

The chemical composition of the analyzed Al–12Si–CuNiMg matrix alloy is shown in Table 1. The matrix alloy was produced by a high-pressure squeeze-casting process. After that, the alloy was homogenized at 750 K for 1 h, oil quenched to room temperature, retained at an ambient condition for 24 h, and then artificially aged at 513 K for 7.5 h.

The Al_2_O_3_ fiber-reinforced composite was fabricated by the squeeze-casting method. Firstly, a preform, which was a shaped porous assembly of Al_2_O_3_ fibers, was prepared and fixed into a solid mold. Then, the process of mechanical pressure infiltration was conducted, in which the molten aluminum matrix alloy was poured into the mold in the temperature range of 1060–1080 K. The heat treatment of the composite was the same as that of the matrix alloy. The material properties of the matrix alloy and its composite are listed in Table 2.

**Figure 1:**
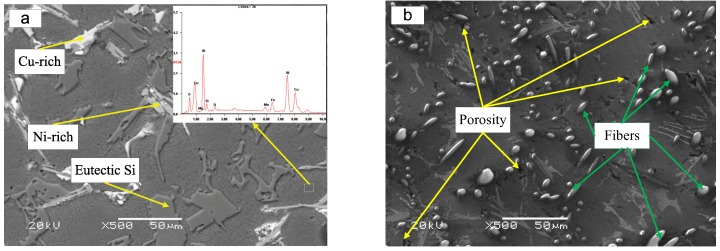
Microstructure of the (**a**) Al–12Si–CuNiMg matrix alloy and (**b**) 17 vol.% Al_2_O_3_ fiber-reinforced composite.

The microstructure of both materials is depicted in Figure 1. It can be seen that the microstructure of the matrix alloy consists of some dendritic grains, eutectic silicon platelets, and fine intermetallic particles (Figure 1a). In addition, for the composite, as shown in Figure 1b, the Al_2_O_3_ fibers were distributed in the matrix, with an estimated diameter of 5 µm and length of about 50–100 µm. The volume fraction of the Al_2_O_3_ fiber is 17%. Furthermore, compared with the matrix alloy, more casting porosities can be found in the composite. In addition, some faveolate defects also exist in the boundary regions between the Al_2_O_3_ fiber and the matrix. The microstructure of the composite was much coarser and less refinable than that of the matrix alloy.

### 2.2. Wear Tests

The dry sliding wear tests for both materials were conducted on a pin-on-disk apparatus (QG-700) under an ambient condition. The matrix alloy and the composite were machined into test pins 5 mm in diameter and 25 mm in height. The counterface disk was made of 0.45% carbon steel with a diameter of 75 mm and thickness of 10 mm. Its Vickers hardness was 240, which was relatively higher than that of the matrix alloy and the composite. The surfaces of pin and disk were polished using grade #1200 emery papers. Mass loss was also measured after cleaning the tested specimen in acetone using precision balance with a sensitivity of 0.0001 g. Furthermore, measured mass loss was converted into volume loss through dividing the cumulative weight loss by the corresponding density.

The tested loads and velocities were 2.5, 5.0, 7.0 N, and 0.25, 0.5 and 1.0 m/s, representing different loading conditions. All wear tests were carried out with a total sliding time of 60 min. In order to determine the friction coefficient, frictional force was continuously monitored during the wear tests. Finally, the worn surfaces of tested specimens were examined by a scanning electron microscope (JSM-5610LV) (JEOL Ltd., Tokyo, Japan) and energy dispersive spectroscopy (EDAX, Mahwah, NJ, USA).

## 3. Results and Discussion

### 3.1. Wear Behavior

**Figure 2:**
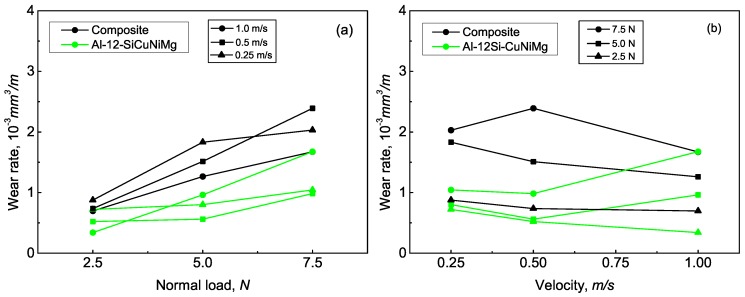
Wear rates of the Al–12Si–CuNiMg matrix alloy and the Al_2_O_3_ fiber-reinforced composite as functions of (**a**) normal load and (**b**) sliding velocity.

The wear rates of the matrix alloy and the composite with reference to different loads and sliding velocities are shown in Figure 2. It can be seen from Figure 2a that the wear rates of both materials increased with increasing load, which is consistent other studies [29,30]. As for the matrix alloy, wear rate under a sliding velocity of 1 m/s linearly increased as the load increased from 2.5 to 7.5 N. Under these circumstances, a higher wear rate was exhibited at 5 and 7.5 N in comparison with that under 0.25 and 0.5 m/s. On the other hand, the influence of sliding velocity on wear rate is relatively ambiguous for the two materials. Under a small load of 2.5 N, the wear rate of the matrix alloy decreased with increasing sliding velocity, as shown in Figure 2b. Nevertheless, wear rate under 5 and 7.5 N first exhibited a slight decrease as sliding velocity increases, then a sharp increase reaching the maximal value at 1 m/s. Therefore, it could be found that the effect of sliding velocity on the wear rate of the matrix alloy is inconsistent under different loading conditions. On the contrary, the relation between wear rate and sliding velocity for the composite is coherent since the wear rate almost continuously decreased as sliding velocity increased except for one point at 0.5 m/s and 7.5 N.

It is of great importance to be noted that the wear rate of the composite was significantly higher than that of the matrix alloy under the same loading conditions. This indicates that the wear resistance of the composite decreased with the addition of Al_2_O_3_ fibers under the present testing conditions. Generally, the addition of Al_2_O_3_ fibers increases the hardness and load-bearing capacity of the composite, which is beneficial for enhancing wear resistance. At the same time, the wear resistance of the composite could also be weakened to some degree by adding Al_2_O_3_ fibers in the matrix [7,21], when the deteriorative effects of the fibers counteract their beneficial effects. Regarding the analyzed 17 vol.% Al_2_O_3_ fiber-reinforced composite, from the macroscopical perspective, the first possible reason for decreased wear resistance could be attributed to the high-volume fraction of Al_2_O_3_ fibers. In the study by Iwai et al., unfavorable influence of high content Al_2_O_3_ fibers on the wear resistance of aluminum matrix composite was found [19]. The dual effect of Al_2_O_3_ fibers on wear resistance was also explored. It was concluded that the optimal volume fraction of the Al_2_O_3_ fiber in improving wear resistance was 12% for the Al–12Si alloys [3]. As for the studied composite, in order to achieve optimal fatigue resistance under high temperatures [26], the content of Al_2_O_3_ fibers was selected as 17%. Apparently, the volume fraction exceeded the optimal value corresponding to wear resistance. Under the circumstances, the deleterious effects produced by the Al_2_O_3_ fibers significantly decreased the wear resistance of the composite.

In addition, from a microscopic perspective, casting porosities in the composite are another factor accounting for decreased wear resistance. As shown in Figure 1b, more casting porosities and faveolate defects have existed in the composite. The appearance of these casting defects increased the tendency of crack initiation and propagation at the Al_2_O_3_/matrix interface during wear tests [18]. The study by Abdulhaqq concluded that the existence of casting porosity increases the actual contact area and subsurface crack propagation at the interface [22]. In conclusion, compared with the matrix alloy, the decreased wear resistance of the composite could be attributed to the high content of Al_2_O_3_ fibers and casting porosities.

### 3.2. Friction Behavior

**Figure 3:**
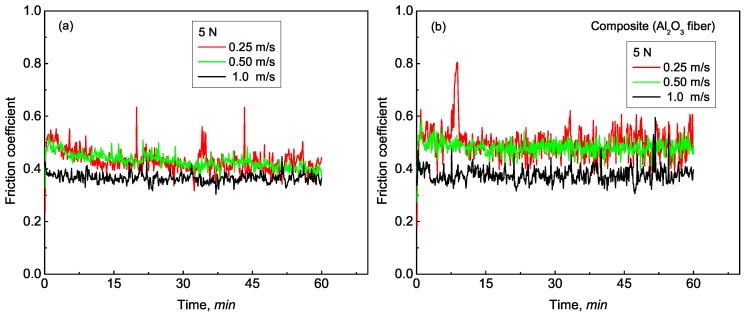
Typical friction-coefficient curves vs. testing time of (**a**) the Al–12Si–CuNiMg matrix alloy under applied load of 5 N and (**b**) the Al_2_O_3_ fiber-reinforced composite under applied load of 5 N.

The friction behavior of both materials corresponding to different loads and sliding velocities was investigated. Figure 3 shows the curves of friction coefficient as a function of sliding time under a load of 5 N. For the analyzed matrix alloy and the composite, it could be observed that, under a constant load, friction coefficient decreases with increasing sliding velocity. After a short stage of initial rise, relatively stabilized fluctuation about a mean value was established for friction coefficients. For both materials, it could also be found that fluctuation amplitude decreased as sliding velocity increased, which indicates that a more stable wear state was reached as sliding velocity increased. Moreover, compared with that of the matrix alloy, the friction coefficient of the composite exhibited a higher fluctuation amplitude under the same sliding velocity, representing a more serious contact and wear state within the composite/disk counterface. The friction coefficient of the matrix alloy also exhibited a slight decrease as sliding time increased, especially at the sliding velocities of 0.25 and 0.5 m/s, which can be attributed to oxide generation by friction heat and surface-roughness refinement resulting in weak junctions within the counterface [22]. Variations of friction coefficients under other loads were almost the same as that presented in Figure 3.

The decrease of the friction coefficient with increasing sliding velocity could be properly attributed to the following reasons. On the one hand, increased sliding velocity reduces adhesion within the counterface due to increased oxidation in the localization [20], since more friction heat is generated as sliding velocity increases. On the other hand, increased sliding velocity reduces the time needed to form a cold junction at the interface between wear pin and disk, which, in turn, decreases the shear force required to cut off the weld joints at the interface.

**Figure 4:**
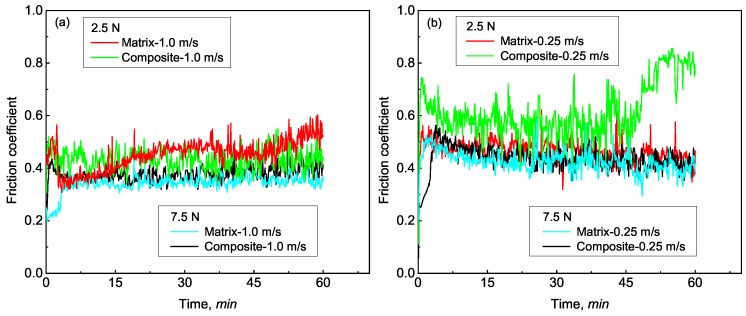
Typical friction-coefficient curves vs. testing time of the Al–12Si–CuNiMg matrix alloy and the Al_2_O_3_ fiber-reinforced composite corresponding to applied loads of 2.5 and 7.5 N under sliding velocities of (**a**) 1.0 m/s and (**b**) 0.25 m/s.

Besides sliding velocity, the influence of load on the friction coefficient was also analyzed. In Figure 4, variations of friction coefficients with a sliding time under loads of 2.5 and 7.5 N are presented. From Figure 4, it can be observed that the friction coefficient of the matrix alloy and the composite decreased as load increased. A similar decrease in friction coefficient was also reported in other aluminum matrix composites [3,18,22]. The decrease of friction coefficient is mainly caused by the increase of surface temperature. Under a high load, the formation of local oxidation layer is promoted resulting in less contact area on the pin surfaces [17]. Moreover, it could be seen that the friction coefficient of the matrix alloy was lower than that of the composite except for the friction coefficient under 1.0 m/s and 2.5 N. The higher friction coefficient of the composite is inconsistent with the results reported in published studies [17,20]. The higher friction coefficient of the composite could be attributed to the formation of tribofilm at the interface between wear pin and disk. When the load impacting on a specific Al_2_O_3_ fiber exceeds its flexural strength, the fiber becomes fragmented and entrapped within the interface, acting as asperities at the interface [31]. Under these circumstances, surface roughness increases over the composite/disk interface, resulting in higher friction force and coefficient.

### 3.3. Worn-Surface Analysis

**Figure 5:**
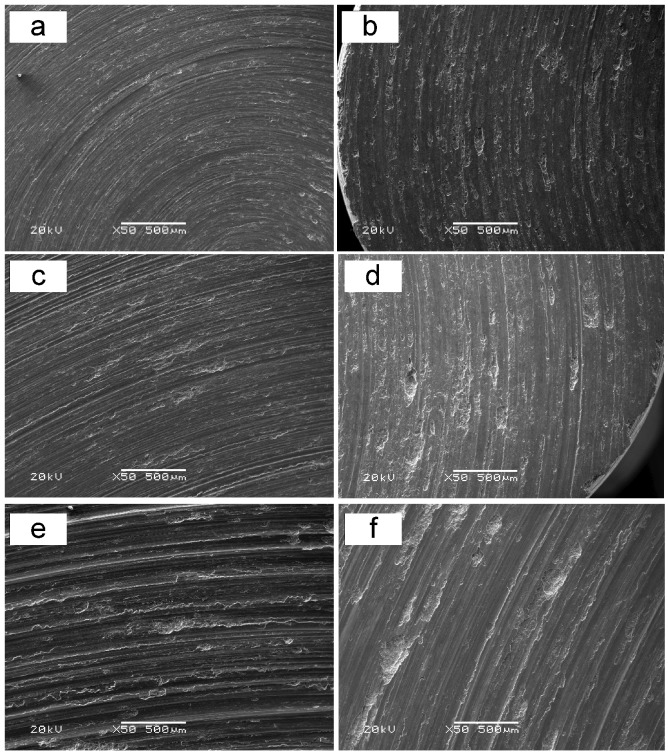
Scanning electron micrographs of worn surfaces under a load of 5 N and sliding velocities from 0.25 to 1.0 m/s. (**a**,**c**,**e**) Worn surfaces of Al–12Si–CuNiMg matrix alloy at 0.25, 0.50, and 1.0 m/s, respectively. (**b**,**d**,**f**) Worn surfaces of Al_2_O_3_ fiber-reinforced composite at 0.25, 0.50, and 1.0 m/s, respectively.

In order to investigate the wear mechanisms, SEM analysis of the worn surface was conducted. The scanning electron micrographs of worn surfaces under a load of 5 N and sliding velocities from 0.25 to 1.0 m/s are presented in Figure 5a–f. As shown in Figure 5a,c,e, many grooves, scratches, and delamination pits could be observed on the worn surfaces of the matrix alloy. Furthermore, the depth and width of grooves significantly increased as sliding velocity increased. The delamination pit is a clear and direct sign of wear loss. The depth and width of grooves generally imply the amount of material removed from the specimen surface [30]. Increased depth and width indicate a higher wear rate at higher sliding velocity. The heavy plastic deformation on the groove edges also became more distinct as sliding velocity increased. As sliding velocity increased, friction heat actually increased to some degree, resulting in the enhanced plasticity flow of the material, which contributed to the increased plastic deformation on the groove edges.

On the other hand, with regard to the worn surfaces of the composite, as shown in Figure 5b,d,f, the situation is totally different from that of the matrix alloy. It can be found that the worn surfaces of the composites were characterized by more delamination pits and wider grooves in comparison with the matrix alloy. Moreover, as sliding velocity increased from 0.25 to 1.0 m/s, the amount of delamination significantly decreased, whereas plasticity deformation on groove edges increased. As aforementioned, decreased delamination indicates decreased wear loss. The worn surface of the composite at 0.25 m/s was relatively flat, and shallow grooves were not very distinct. As sliding velocity increased, friction heat increased and plasticity flow of the composite on the counterface was enhanced, which were the reasons for deeper and increased grooves. Overall, from the worn-surface observations, it can be found that the worn surface of the composite was relatively finer at a higher sliding velocity, indicating decreased wear loss, which is consistent with the result shown in Figure 2.

Comparing the worn surfaces of the matrix alloy with that of the composite, it can be seen that the depths of grooves on the composite were relatively shallower and less distinct than those on the matrix alloy. This phenomenon can be ascribed to the higher hardness of the composite restricting plastic deformation. At the same time, delamination pits that exist on the composite surfaces were relatively heavier under all testing conditions. This phenomenon, as mentioned in Section 2.1, can be attributed to the casting porosities in the composite and weak bonding between fibers and the matrix. Under these circumstances, the benefit of the fibers in improving load bearing and solid lubrication is significantly weakened, inducing decreased wear resistance of the composite contrasted with the matrix alloy.

**Figure 6:**
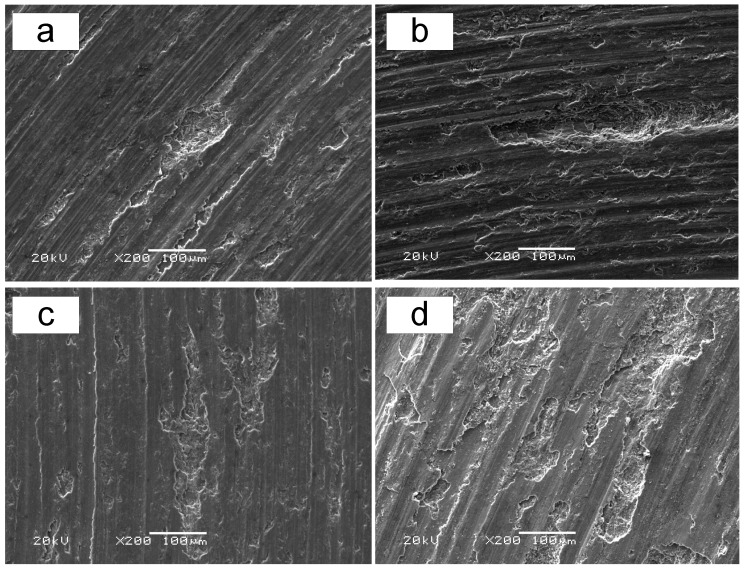
Scanning electron micrographs of worn surfaces under a sliding velocity of 0.50 m/s and loads of 2.5 and 5.0 N. (**a**,**c**) Worn surfaces of Al–12Si–CuNiMg matrix alloy under loads of 2.5 and 5.0 N, respectively. (**b**,**d**) Worn surfaces of Al_2_O_3_ fiber-reinforced composite under loads of 2.5 and 5.0 N, respectively.

The influence of the load on the worn surfaces of both materials is presented in Figure 6. As applied load increased, the areas of delamination significantly increased for both materials. In addition, slight and shallow grooves have been rubbing down due to increased shear force at high load. Furthermore, under the same loading condition, the worn surfaces of the composite contained larger delamination pits and the width and depth of grooves were also much larger, which indicated higher wear loss. From this figure, it can be seen that the increase of load aggravates the generation of delaminations on the counterface, resulting in serious wear loss.

**Figure 7:**
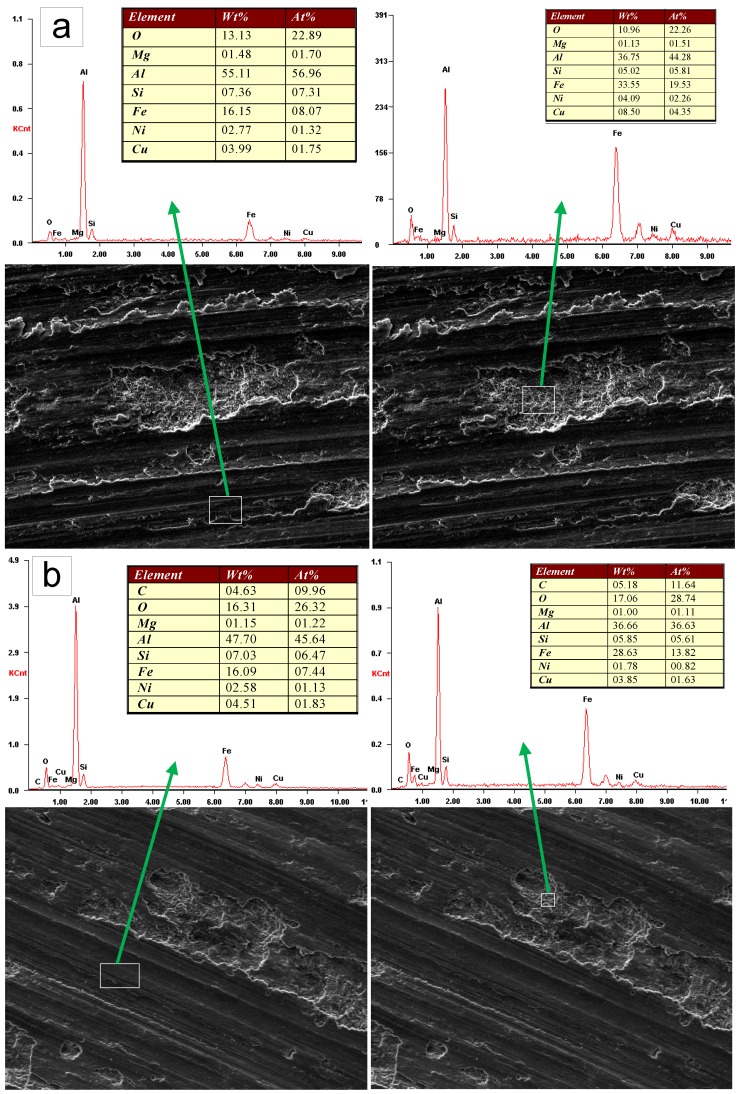
Energy dispersive spectroscopy (EDS) analysis results for the (**a**) Al–12Si–CuNiMg matrix alloy and (**b**) composite under identical wear conditions at 1.0 m/s and 5.0 N, respectively.

EDS analysis of the two materials was conducted as shown in Figure 7. From this figure, it could be observed that an amount of iron appeared on the surfaces of both materials. Furthermore, the iron existed not only on the contact surface between pin and disk, but also on the surface of the delamination pits. The counterpart in the wear tests was made of 0.45% carbon steel; therefore. the iron on the worn surfaces of the pins was transformed from the disk by a mechanism of mechanical alloying [32]. Research by Rosenberger et al. [33] concluded that the iron-transfer layer generated by the adhesion of the materials could inhibit contact between the counterfaces. EDS analysis showed a distinct proof of adhesion wear in both materials. Under these circumstances, considering the foregoing analysis of worn surfaces from which abrasive wear is confirmed, it could be concluded that the dominant wear mechanisms for the matrix alloy and the composite are abrasive wear and adhesion wear.

## 4. Conclusions

(1) Under the present testing conditions, the wear rate of the Al_2_O_3_ fiber-reinforced composite was relatively higher than that of the Al–12Si–CuNiMg matrix alloy. This phenomenon could be attributed to the high content of Al_2_O_3_ fibers and casting porosities in the composite.
(2) The wear rate of the composite decreased with increasing sliding velocity. With regard to the matrix alloy, the wear rate exhibited a distinct increase with increasing sliding velocity.
(3) The friction coefficient of the two materials decreased as sliding velocity increased. Furthermore, the friction coefficient significantly decreased for both materials with increasing load. Compared with that of the matrix alloy, the friction coefficient of the composite was relatively higher due to the formation of tribofilm on the counterface.
(4) Under present testing conditions, the dominant wear mechanisms for both materials were abrasive wear and adhesion wear.

## Figures and Tables

**Table 1:** Chemical composition of analyzed Al–12Si–CuNiMg matrix alloy (in wt.%).

Si	Cu	Ni	Mg	Fe	Mn	Ti	Zn	Al
12.37	5.28	2.67	0.82	0.42	0.20	0.106	0.006	Bal.

**Table 2:** Material properties of Al–12Si–CuNiMg matrix alloy and Al_2_O_3_ fiber-reinforced composite.

Material	Ultimate Tensile Strength MPa	Young’s Modulus GPa	Vickers Hardness HV	Density Kg/m^3^
Matrix alloy	260	80	130	2770
Composite	287	88	160	2790
